# Teledermatology: Its Role in Dermatosurgery

**DOI:** 10.4103/0974-2077.44162

**Published:** 2008

**Authors:** Garehatty Rudrappa Kanthraj

**Affiliations:** *Department of Dermatology, Venereology and Leprosy, Jagadguru Sri Shivarathreshwara University Medical College Hospital, Ramanuja Road, Mysore, Karnataka, India*

**Keywords:** Dermatosurgery, teledermatology, dermatopathology, dermatology

## Abstract

Dermatologic surgery and aesthetic dermatology are rapidly emerging and expanding specialties in India. However, dermatologists practicing surgeries and aesthetics in India represent a highly selected group and are mostly confined to metros. Dermatologists in the peripheral and remote regions need to reach these specialists for the benefit of their patients and teledermatology is an invaluable tool for this purpose. Video-conference, store and forward, Satellite communication, Hybrid teledermatology, mobile teledermatology, Integration model, nurse-led teledermatology, teledermatology focusing difficult-to-manage cases, screening and triage services are the various teledermatology services developed to suit the needs of dermatology care from a distance. Types of teledermatology service, pattern of net work connectivity and purpose of dermatology service are the three cardinal parameters for management of the dermatoses from a distance. This article reviews the literature, and analyzes the possible options available for a teledermatosurgery practice.

## INTRODUCTION

Consultation is seeking an expert opinion. Expert opinion obtained for medical purpose from a distance is called Telemedical consultation. The distance can be between the continents, countries, states, cities or even doctors a few meters apart. World health organization defines telemedicine as practice of health care using interactive audio, visual, and data communications. In 1995 Prednia and co-workers[1] introduced the term ‘Teledermatology’ (TD) and in 1997, Zelickson and Homan[2] first demonstrated video conference teledermatology in their nursing home setting. TD is a subset of telemedicine that incorporates telecommunications technologies (information technology) to deliver dermatology services at a distance. The common principle of TD services is to reach the unreached for dermatology care.

Telemedicine reduces travel, waiting time, treatment cost, minimizes follow-up visits and helps to deliver specialty health care services to remote geographic regions. Dermatology is a Visual specialty; Image is the gold standard for dermatological diagnosis.[3] Chronic disorders that require a long duration of treatment need frequent monitoring and several followup visits. It involves travel expenses, and also a prolonged waiting time. These the above circumstances have made dermatology an ideal specialty for telemedical applications and it is not suprising that dermatologists have been early adopters for Tele medical applications.[4]

TD delivers screening and triage services in Melanoma and pigmented skin lesions.[3] TD can be used for screening, triage and select patients for dermatosurgery practice. TD delivers treatment and follow-up care in leg ulcers and to discuss difficult -to-manage cases like the Inflammatory and neoplastic conditions[5] from worldwide experts and Education of health care professionals and patients. It is observed to achieve 81% concordance for exchanging opinions on challenging inflammatory and skin neoplasm’s.[5]

## CLASSIFICATION

**Figure 1:**
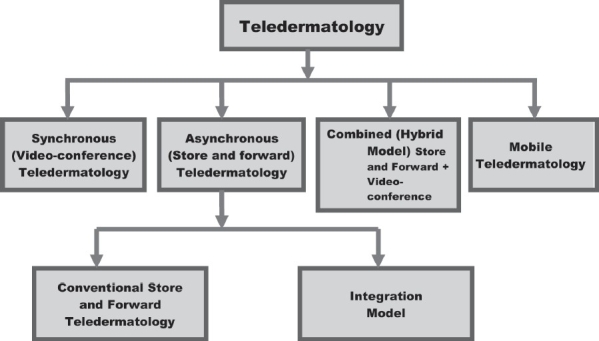
Various teledematological tools

Different teledermatological tools available are [Figure 1]:

### Real time or videoconference

Video consultation uses video-conferencing equipment to connect the patient, often with their general practitioner (GP) or nurse present, with a distant consultant. The evaluation of diagnostic accuracy varies between 67-80% compared to face-to-face consultation.[6] Initial studies on economic evaluation of interactive teledermatology compared to face-to-face consultation considered video conference to be expensive. However more recent studies[7] have confirmed it to be economical. This is due to the improved technology and decrease in the hardware cost. Table 1 summarizes the various types of videoconference (VC) available for practice.

**Table 1:** Type of videoconference, their components and cost-effectiveness while setting a video conference teledermatology center[16]

Type	Components	Cost
Stand-alone VC	Video codec with built in camera (pan, tilt and zoom), built in micro phone and audio-video interfaces to connect line/network interface ISDN/LAN	Expensive
PC based VC with PC add-on card codec	PC, web camera with built in microphone with audio/video output to connect ISDN/LAN	Moderately expensive
PC based VC with In-Camera codec*	Built in video-camera is connected to the PC through USB port.	Less expensive
PC based VC Using web camera And software*	Video codec function (audio-video compression and data formatting) is done using software loaded on PC.	Cheapest
	A web camera is connected to PC using a USB port.	

* To connect to multimedia projector and achieve large display VGA/XVGA out put of the PC may be used. (Reproduced with permission Kanthraj GR and Srinivas CR. Ind J Dermatol Venerol Leprol 2007; 73:5-12.)

Satellite communication net work (Satcom) is an Indian space research organization (ISRO) initiative to reach the unreached and inaccessible remote geographic regions where connectivity cannot be established is achieved through satellite connectivity.[8] Skin camps are organized by mounting SATCOM on a bus or a van that travels to those remote geographic regions where Integrated services digital network (ISDN) connection cannot be established and establishes satellite net work connectivity with a tertiary center[9] and delivers dermatology care.

### Store and forward system

The store and forward (SAF) stores patient data (digital images, clinical and demographic information) sent by GP’s in an electronic medium for future access by consultants in referral centers to deliver the quality health care in remote geographic regions. SAF involves transmission of digital images and asynchronous evaluation is practiced. Simultaneous presence of the health care professional is not required. About 80-90% of dermatological conditions may be diagnosed by SAF TD.[10] It is the most commonly used technology. There is 60–80% total agreement and 70-90% partial agreement when in person diagnosis is compared to both real time (synchronous) and SAF TD.[10] Various feasibility studies on SAF TD are summarized in Table 2 and it has been found to be cheap, easy to set up and practice.

Low cost electronic equipments, quick electronic transfer of high quality digital images and universal access to the health care workers enhance the practice of SAF. SAF uses digital camera with an average 640 × 480 pixels image resolution. Different diagnosis agreement rates (of 68%,[11] 89%,[12] 58%[13] and 48%[14]) have been documented in various studies. The images are rapidly transferred[6] and stored in JPEG (Joint photographers expert group; http://www.jpeg.org) format using the internet. Poor image quality and lack of referral proforma data may lead to poor agreement.[14] Telemedical wound care and follow up uses digital camera with a good agreement to face-to-face, which is regarded as the Gold standard.[15]

A referral hospital can have a fixed time on a day depending on the caseload for a teledermatology clinic to prevent unnecessary delay. General practitioner can send history with photograph well in advance. Relevant discussions are made, as both ends are prepared. Patient, general practitioners or nurse available in the given period, interacts by e-chat or web cam or voice mail for any clarifications required from the consultant. This approach has been recommended while offering periodic follow-up care.[16] SAF provides patient, referring clinician and dermatologist satisfaction.[7] Referring clinician has an additional advantage of educational benefit.[7]

### Data transmission medium: The Internet, Wi-Fi and Wi-Max

Digital lines to enhance data transmission are called ‘Integrated services digital network’ (ISDN). Increase in the number of ISDN line increases the data carrying capacity or bandwidth from 128 kbps (one ISDN line) to 256 and 384 kbps (two and three ISDN lines) respectively.[6]

Wi-fi[16] and Wi-Max[16] are super speed wireless network connections that enable high-speed transmission of the data. Wi-Fi (wireless fidelity) enables wireless local area network (WLAN) connections to mobile devices, digital camera, PC computers and personal digital assistants.[16] There devices are with Wi-Fi built in, while others require adding a Wi-Fi network card. Wi-fi uses radio-waves, radio transmitters called routers with receivers (access points / hot spots). Superior to Wi-Fi is Wi-Max (worldwide interoperability for micro wave access). It is a broadband wireless access technology providing super speed Internet access at 70mbit/s. Images may be received in large number by referral hospital during a clinical trial or teleconsultation. Speed of the transfer medium is important for rapid and easy retrieval of large data. Therefore, a telemedical center should have Wi-Fi or Wi-Max installation for Quality and Speed of data transmission.[16]

### Hybrid model

The combination of SAF TD in the first step, followed by VC TD in the second step is called Hybrid TD.[17,18] It saves time, clarifies doubts and avoids misinterpretation from both the ends. This process achieves best physician and patient satisfaction.

### Mobile or cellular teledermatology

Portable devices like cellular phones and personal digital assistants provides inbuilt camera to capture digital images, computing, and net working features to deliver dermatology care at a distance. They provide immediate image access and direct interaction and it is possible to obtain clarification. Periodic evaluation of leg ulcers and skin images using cellular phones and Personal digital assistants are practiced. Quality and speed of image transmission is no longer an obstacle. Melanoma screening with cellular phones using mobile teledermocscopy revealed a diagnostic agreement of 90% compared to face-to-face consultation.[19]

Cellular phones[20,21] and Personal digital assistants[22,23] allow taking good quality images and sending them to the expert from remote geographic regions via a wireless network e.g., global system for mobiles (GSM) and universal mobile telecommunication system (UMTS). New generation cellular phones allow to take good quality images and transmit them directly to other cellular phones (via multimedia messages) and computers (via e-mail or blue tooth-wireless connection) the diagnosis agreement is 82% compared to face-to-face consultation.[20] A feasibility study[20] confirmed the importance of cellular phone in telemedical wound care. Transfer of skin images using cellular phones with a diagnosis agreement of 70% is documented.[21] Various feasibility studies on mobile TD are summarized in Table 2.

**Table 2:** Feasibility studies involving store and forward and mobile (cellular) teledermatology practice

Author/Year	Contribution	Device	Model	Charge couple device reskolution (mege pixles)	Image resolution (pixles)	Storage (JPEG)KB	Diagnosis agreement* %
Whited *et al*.[11], 1999	Store and forward of skin lesions	Digital camera	-	-	1280×1000		68
High *et al*.[12], 2000	Store and forward of skin lesions	Digital camera	Sony DSC – F1	-	640×480	-	89
			Sony Corporation Newyork				
Tucker *et al*.[13], 2005	Digital imaging of skin lesions for teleconsultation	Digital camera	Fuji-film MX-1700	-	640×480	32MB	58
Mahendran *et al*.[14], 2005	Digital imaging and teleconsultation of skin malignancies	Digital camera	Coolpix 950 Nikon Corporation	-	1200×1600	-	48
Salmhofer *et al*.[15], 2005	Digital camera in wound teleconsultation	Digital camera	Coolpix995 Nikon Corporation	3.3	2048×1536	1500	87
Braun *et al*.[20], 2005	Cellular phone in telemedical wound care	Cellular phone	Nokia 7650 Espoo, Finland	-	640×480	15-22	82
Massone *et al*.[21], 2005	Cellular Phone in teledermatology	Cellular phone	Nokia 7650 Espoo, Finland	-	640×480	13-35	70
Massone *et al*.[22], 2006	Personal digital assistants in teledermatology	Personal digital assisstants	Sony Clie PEG – NZ90, Tokyo, Japan	2	1200×1600	829-989	79

* Compared to face to face consultation (Gold Standard)

### Personal digital assistants

Laptops, hand held computers are convenient to handle and offer combined features like camera, computing, and networking are called personal digital assistants.[22,23] They are convenient for health care professionals to capture and transfer the images. Massone *et al*,[22] demonstrated the importance of personal digital assistants in teledermatology with a 79% diagnosis agreement.

Diagnostic agreement is low for cutaneous malignancies when there is poor quality of images, and lack of referral proforma data.[14] Recent studies[24] have proved that digital photography gives such a high image quality, that a neoplastic lesion that cannot be diagnosed with a high quality digital image, rarely could be diagnosed with face -to -face consultation.[24] Advances in digital imaging and internet solutions have overcome technical limitations.[3] Teleconsultation applied as a screening method for malignant tumors is a useful technique that can be incorporated in dermatology for day- to- day practice.[24]

### Integration model

Health care professionals in rural areas are the’ eyes’ for the expert. For the expert to guide them, deliver follow up and monitor the progress they should have a periodic audit of visual parameters and dimensions.This has been demonstrated in the treatment and follow up of leg ulcers. Various authors[25–31] have demonstrated the effectiveness of computerized measurements of leg ulcers to monitor therauptic assessment.

**Figure 2:**
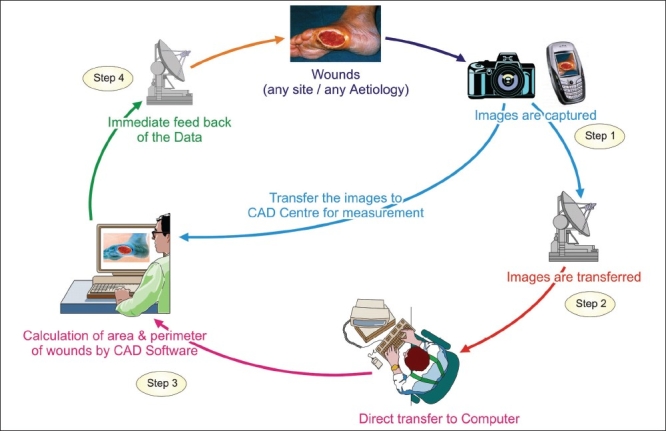
The integration model to capture, transfer, measure and follow-up of skin lesions to deliver SAF teledermatology care[32] (Reproduced with permission from American Medical Association, Kanthraj GR. Arch Dermatol 2005;141:1470-71)

The systematic functional integration of electronic devices and software to capture, transfer, store, measure and deliver follow-up care is the principle of integration model and has been used effectively for leg ulcers in remote geographic regions.[32] It is illustrated in the [Figure 2]. In step 1 close up image of the ulcer with the surrounding skin is suitable for teleconsultation.[6] The images are captured, irrespective of its site either by digital or cellular phones camera [Figure 2]. In step 2 images are transferred via e-mail. In Step 3 calculations of wound margins (area and perimeter) is done by Computer Aided Design software (CAD). In Step 4 the software professional delivers the result. Periodic evaluation is performed. Bizarre shaped lesions at any site are measured accurately and stored. It is useful for forensic experts to reproduce at the time of expert evidence.

Immediate access of visual parameters and measurement of lesions are achieved. Routine follow- up care in a remote area under close supervision of higher center is performed. Computerized measurements are rapid, easy, and precise and suited for SAF TD.[16,32] This approach enables diagnosis, management, and periodic assessment of leg ulcers and delivers follow-up care to achieve physician and Patient satisfaction. Repigmented vitiligo can be serially monitored after medical or surgical treatment.[26] Serial monitoring of the images and determination of their dimensions and percentage of pigmentation documents the progress of re-pigmentation.

The system automatically plots wound healing regression or vitiligo repigmentation on the computerized graph. Rapid capture, transfer and calculation with negligible human intervention minimizing inter observer and intra observer variations are achieved.[32] The programmer does the calculation and provides the results to dermatologists at telemedical center. Computer professionals process the large data without any additional burden to the experts. All health care professionals at a tertiary centre utilize the software in a centralized location maximizing SAF telemedical center’s utility and generalizability.[27]

## APPLICATIONS OF TELEDERMATOLOGY

### Teledermatological monitoring of leg ulcers

SAF and mobile TD play a key role in leg ulcer management. It is a chronic disorder that requires frequent and periodic monitoring. Patient needs frequent visits from a Long distance incurring huge costs. It can be more distressing in elderly as leg ulcer is known to occur more frequently requiring prolonged waiting time. HCP needs to be trained for digital photography, uploading, web application and wound care. 90% of images for consultation are excellent.[33] Good response is observed in 70% of cases with a good patient and physician satisfaction.[33] SAF and mobile TD hold a great potential for long term wound care along with the Co-operation with home care nurses.[25] Leg ulcers are monitored for slough, necrosis and granulation tissue formation.[15] In a recent study the feasibility and acceptance of teledermatology for wound management has been demonstrated.[34] Consultant personally examined, assessed, classified and recommended the medications for the ulcers in the initial visit. Follow up visits were done by the home care nurses using SAF TD.. In 90% of teleconsultations qualities of images were good with good physician and patient satisfaction.[33]

### Teledermatopathology

Teledermatopathology is an important area for application of telemedicinal tools.[35–37] Teledermatopathology involves transmission of images from distant locations to consulting dermatopathologists. It is of relevance to the Indian scenario where there are only a few dermatopathologists and even general histopathologists are available only in citiis. Teledermatopathology is achieved by a) video- image (dynamic) analysis b) store and forward (static) c) and virtual slide system (VSS).[35]

VSS is a recently developed technology wherein using a robotic microscope, any field of the specimen is magnified at the discretion of the dermato-pathologist. VSS stores the images on a virtual slide server available on the web.[36] VSS is being used for the diagnosis of pigmented skin lesions, difficult- to-manage cases and inflammatory skin diseases. Though more expensive than the store and forward system, VSS represents the future in this discipline. A study[36] investigated the role of teledermatopathology by VSS in forty-six biopsy specimens from inflammatory skin diseases. Telediagnoses agreed with gold standard and conventional diagnosis with an average of 73% and 74%, respectively.[36] Complete concordance among all teleconsultants with gold standard and conventional diagnosis was found in 20% of the cases. The study concluded that the system is not fully suitable for diagnosis of inflammatory dermatoses, as the diagnosis and interpretation often depends on availability of complete clinical data and because of the intrinsic difficulties in diagnosis of such diseases. This is of particular relevance to Indian situation where most biopsies are performed for inflammatory diseases and where skin cancer is less prevalent. Recent concept of Hybrid teledermatopthology[37] incorporates the advantages of both dynamic and virtual slide system and over comes the individual shortcomings. It consists of motorized microscopes with remote control and scanner for slide digitization.

### Teledermatology in Cutaneous aesthetic surgery

SAF can be used to screen and determine the suitability of the lesion for treatment by dermatosurgery. Some examples include conditions such as keloids, hemangiomas, scars, vitiligo lesions for grafting, hirsutism for laser assisted hair reduction, ageing changes of skin and images of scalp for hair transplantation etc. VC can also be used for pre-surgery counseling for aesthetic procedures. VC increases patient satisfaction as patient directly interacts with the aesthetic surgeon for any clarifications. Selected centers can have Hybrid teledermatology to screen patients for procedures and counseling for aesthetic procedure. Mobile teledermatology is used to screen and deliver follow-up care after aesthetic surgery. Integration model[32] finds its application in objective assessment after medical or surgical treatment of vitiligo and leg ulcers. Types of tedermatology service, purpose and area of application and their potential use in aesthetic surgery are summarized in Table 3.

**Table 3:** Types of teledermatology service, purpose, area of application and their potential use in dermatosurgery

Type of teledermatology service	Purpose	Dermatoses/Area of application
Hybrid model (Combination)	Incorporates the advantages of both synchronous and asynchronous teledermatology	Routine teledermatology service and follow-up care
Conventional store and forward teledermatology	Screening	To determine the suitability for a dermto-surgery
Video-conference teledermatology	Counseling and Education	Pre surgery counseling
Integration model	Follow-up care after medical or surgical treatment	Leg ulcers and vitiligo
Mobile or cellular teledermatology	Sreening and follow-up care after dermatosurgeries	Leg ulcers, vitiligo or any dermatosurgery procedure

### Nurse-led teledermatology service

Training of Health care professionals for TD practice at all levels in basics of dermatology including computer knowledge, art of counseling, history taking skills, filling the Performa, photography and Video clips. Administration of intralesional steroids, training for skin biopsies, removal of skin tags, cryosurgery and other simple dermatosurgical techniques are taught.[38] Interprofessional collaborative working environment to share the expertise in decision making is promoted. A nurse advice the management guided by consultants, motivates and delivers for follow-up care. They understand the physical, psychological and social effects of skin disease.[39]

### Counseling

Preparing the patient for TD practice by educating the dermatoses, the technology adopted and its limitations involved in delivering dermatology care. Medical reimbursement equivalent to that of the traditional face-to-face examination in an approved rural health care setting are practiced.[18]

### Imaging and law

Photographs form important medico-legal evidence and play a vital role in maintenance of the dermatology records.[40] It has its special significance as digital images are used to capture, store, measure, transfer and deliver follow up care. The image measurement is important for periodic follow-up care and medico-legal expert opinion. US Federal courts have ruled that digital images can furnish sufficient medical data to provide dermatological care.[40] Preservation of privacy and confidentiality of digital images in the era of teledermatology is important.[3,41] In difficult- to-manage Case or in doubt, a dermatologist can be sued for a wrong diagnosis. “One cannot take shelter on the pretext of a teledermatology consultation.” A principle of traditional consultation applies to TD care.[6] In case of doubt a dermatologist should call for face-to-face examination and investigates on priority. They should obtain written consent from patient to store and forward the images. Confidentiality of Images has to be maintained.[6]

### FUTURE DIRECTIONS

Health access barriers, poverty, large geographic regions and deficiency of dermatologists in rural regions have increased the needs of the TD service in India. A Proper reimbursement and insurance policies need to be implemented for teledermatology consultation and surgery. Training the existing nurses for digital photography, internet applications and computing needs to be implemented. Mobile TD finds its application and active survey is carried out by HCP using cellular phones to screen the dermatoses. Teleconsultation is an effective alternative and should be considered when a service is under pressure.[42] Incorporation of telemedicine and nurse led telemedicine effectively in rural India can enhance delivery of health care. Academic bodies have to introduce telemedicine education in medical and nursing training. House surgery period in undergraduate education should be made to used train telemedicine and thereyby offer service to rural India.

## CONCLUSION

TD research is progressing in an arithmetic ratio (in additions) while advancement in information technology is progressing in geometric ratio (in multiples).[16] Dermatosugoens need to explore the feasibility of technology application in the interest of the patient and conduct studies. They need to work along with their respective Government, Ministry of Health and national health services of their regions to formulate clinical, technical and administrative standards to facilitate expansion of teledermatology in practice and introduce it as a curriculum in medical education. There is need to to accelerate research and adopt innovative techniques to deliver quality health care to remote geographic regions and achieve the goal to reach the unreached.
